# Supplementary material for: Anesthesia videos in geriatric and elderly patients on YouTube: content, quality, reliability, and usefulness assessment
Source: PeerJ. 2025 Apr 17;13:e19280. doi: 10.7717/peerj.19280 (PMC12009561; doi:10.7717/peerj.19280)
Supplement: Supplemental Information 2 — Anesthesia Videos for Geriatric and Elderly Patients on YouTube: Quality, Reliability, Content, and Usefulness Assessm… [file peerj-13-19280-s002.pdf]

Turan Evran

Anesthesia Videos for Geriatric and Elderly Patients on YouTube: Quality, Reliability, Content, and Usefulness Assessm...

Quick Submit

Quick Submit

Pamukkale Üniversitesi

Belge Ayrıntıları

Gönderi Kimliği

trn:oid:::1:3011225000

Gönderi Tarihi

17 Eyl 2024 16:02 GMT+3

İndirme Tarihi

17 Eyl 2024 16:11 GMT+3

Dosya Adı

manuscript_file.docx

Dosya Boyutu

59.9 KB

16 Sayfa

6.429 Sözcük

37.356 Karakter

15% Genel Benzerlik

Her veri tabanı için çakışan kaynaklar da dâhil tüm eşleşmelerin kombine toplamı.

Rapordan Filtrelenen

- Bibliyografya

Eşleşme Grupları

63 Atıf ya da Alıntı Yapılmamış 13%
Ne metin içi atıf ne de tırnak işareti içeren eşleşmeler

7 Eksik Alıntılar 2%
Kaynak materyale hâlâ çok benzeyen eşleşmeler

0 Eksik Atıf 0%
Tırnak işaretleri olan ancak metin içi atıfları olmayan eşleşmeler

0 Atıf Yapılan ve Alıntılanan 0%
Metin içi atıf içeren ama tırnak işareti içermeyen eşleşmeler

Ön Sıradaki Kaynaklar

9% İnternet kaynakları
13% Yayınlar
1% Gönderilen çalışmalar (Öğrenci Makaleleri)

Bütünlük Bayrakları

İnceleme için 0 Bütünlük Bayrağı

Herhangi bir şüpheli metin manipülasyonu belirlenmedi.

Sistemimizin algoritmaları bir belgede, onu normal bir gönderiden ayırabilecek her türlü tutarsızlığı derinlemesine inceler. Tuhaf bir şey fark edersek incelemeniz için bayrak ekleriz.

Bir Bayrak mutlaka bir sorun olduğunu göstermez. Ancak daha fazla inceleme için dikkatinizi vermenizi öneririz.

Eşleşme Grupları

- **63** Atıf ya da Alıntı Yapılmamış 13%
  Ne metin içi atıf ne de tırnak işareti içeren eşleşmeler
- **7** Eksik Alıntılar 2%
  Kaynak materyale hâlâ çok benzeyen eşleşmeler
- **0** Eksik Atıf 0%
  Tırnak işaretleri olan ancak metin içi atıfları olmayan eşleşmeler
- **0** Atıf Yapılan ve Alıntılanan 0%
  Metin içi atıf içeren ama tırnak işareti içermeyen eşleşmeler

Ön Sıradaki Kaynaklar

9% İnternet kaynakları
13% Yayınlar
1% Gönderilen çalışmalar (Öğrenci Makaleleri)

Ön Sıradaki Kaynaklar

Gönderi içinde en yüksek eşleşme sayısına sahip kaynaklar. Çakışan kaynaklar görüntülenmeyecektir.

#	Tür	Kaynak	%
1	İnternet	www.ncbi.nlm.nih.gov	3%
2	Yayın	Sena Tolu, Ozan Volkan Yurdakul, Betul Basaran, Aylin Rezvani. "English-languag...	1%
3	İnternet	turkarchotolaryngol.net	1%
4	Yayın	Xin-Yuan Han, Xue-Ling Wang, Jin Zhang, Xue-Lei Gong, Li-Juan Kan, Jie-Hong Wei,...	1%
5	İnternet	www.revistagastroenterologiamexico.org	0%
6	Yayın	Maria Luiza Silva Aragão, Ianne Moreira Vieira, Rafael Domingos Almeida Durand...	0%
7	Yayın	Elif Tarihci Cakmak, Serpil Celik. "Characteristics, reliability, and quality of YouTu...	0%
8	Yayın	Linfang Zhu, Yang Liu, Fengxue Yang, Shaobin Yu, Ping Fu, Huaihong Yuan. "Asse...	0%
9	İnternet	www.e-cep.org	0%
10	Yayın	Maria Pia Tropeano, Beatrice Claudia Bono, Giovanni Battista Lasio, Zefferino Ros...	0%

11 Yayın

Mesut Berkan Duran, Yalcin Kizilkan. "Quality analysis of testicular cancer videos ... 0%

12 Yayın

Gurinderjeet Singh, Harshita Chandrashekhar Devadiga, Rashmi Prakash, Dakshi... 0%

13 Yayın

Yeliz Culha, Mehmet Gokhan Culha, Rengin Acaroglu. "Evaluation of YouTube Vid... 0%

14 İnternet

www.mdpi.com 0%

15 Yayın

Aydın KESKİNRÜZGAR, Günay YAPICI YAVUZ, Elif ACIBADEM. "Reliability of Inform... 0%

16 Yayın

Dilek Demir, Murat Bektas. "The effect of an obesity prevention program on child... 0%

17 Yayın

Hyunjin Cho, Feiyan Yi, Sukhee Ahn. "Quality evaluation of pregnancy-related mo... 0%

18 Yayın

Sevda Korkut, Ayla Ünsal, Ali Kaplan. "Comparison of Turkish and English YouTub... 0%

19 Yayın

Sevgi Koroglu, Gulgun Durat. "Misophonia: A self-report-based scale developmen... 0%

20 Yayın

Xiang Zhang, Yi Yang, Yi-Wei Shen, Ke-Rui Zhang, Li-Tai Ma, Chen Ding, Bei-Yu Wa... 0%

21 Yayın

Hakan Silek, Ozgur Bilgin Topcuoglu. "Analysis of YouTube videos as a source of i... 0%

22 Yayın

Michael Wong, Bhumit Desai, Michele Bautista, Ohmin Kwon, Nicholas Kolodychu... 0%

23 Yayın

Narayan Prasad Belbase, Sagar Khatiwada, Nishnata Koirala, Hari Prasad Upadhy... 0%

24 Yayın

Ren-Chun Du, Yang Zhang, Meng-Hui Wang, Nong-Hua Lu, Yi Hu. " and Bilibili as ... 0%

25 Yayın

Zeyang Chen, Shaorong Pan, Meng Zhou, Xin Wang. "Evaluation of the quality an... 0%

26 İnternet

www.dovepress.com 0%

27 İnternet

www.ijrcog.org 0%

28 Yayın

Anita R. Iskandar, Benchun Miao, Xinli Li, Kang-Quan Hu, Chun Liu, Xiang-Dong W... 0%

29 Yayın

Binhan Aktas, Doruk Demirel, Ferhat Celikkaleli, Suleyman Bulut, Emrah Ozgur, Y... 0%

30 Yayın

Halil Uzundal, Türker Soydaş, Selman Ünal, Duygu Ercan Uzundal. "Quality and Re... 0%

31 Yayın

Ying C. Ku, Lianne Mulvihill, Jacob Lammers, Mazen Al-Malak et al. "Comparing th... 0%

32 İnternet

mededu.jmir.org 0%

33 İnternet

www.lenus.ie 0%

34 Yayın

De-Ping Han, Cai-Qian Gou, Xin-Mian Ren. "Predictive utility of the Rockall scoring... 0%

35 Yayın

Georges Ayoub, Elie Chalhoub, Ghassan Sleilaty, Hampig Raphael Kourie. "YouTu... 0%

36 Yayın

Muhammer Ergenç, Tevfik K. Uprak. " YouTube as a source of information on : Co... 0%

37 İnternet

research-repository.griffith.edu.au 0%

38 İnternet

www.thieme-connect.de 0%

39 Yayın

İlhan Uzel, Behrang Ghabchi, Ayşe Akalın, Ece Eden. "YouTube as an information ... 0%

40 Yayın

Adil Emre Gezer, Veysi Siber, Merve Yazla, Emine Sarcan et al. "Compliance of the ... 0%

41 Yayın

Anna Kiss, Sándor Soós, Ágoston Temesi, Brigitta Unger-Plasek, Zoltán Lakner, Or... 0%

42 Yayın

H.Bahadir Gokcen, Gurkan Gumussuyu. "A Quality Analysis of Disc Herniation Vid... 0%

43 İnternet

bmcpublichealth.biomedcentral.com 0%

44 İnternet

gcris.ieu.edu.tr 0%

45 İnternet

siuj.org 0%

46 İnternet

www.cyprusjmedsci.com 0%

47 İnternet

www.journal-jmsr.net 0%

48 İnternet

www.science.gov 0%

49 İnternet

www.scilit.net 0%

50 Yayın

asli cetinkaya yaprak, Çisil Erkan Pota. "Assesment of the quality of information p... 0%

Anesthesia Videos for Geriatric and Elderly Patients on YouTube: Quality, Reliability, Content, and Usefulness Assessment

Abstract:

Purpose:
The proportion of elderly individuals requiring surgical interventions is increasing globally, constituting about half of the surgical population in developed countries. Although accessing information online is relatively easy, the ability of users to effectively evaluate the quality and accuracy of such information remains limited. This study aimed to assess the quality, reliability, content, and usefulness of YouTube videos related to anesthesia in geriatric and elderly patients.

Methods:
Using Google Trends, the most popular search terms in the past five years, "geriatric anesthesia" and "anesthesia in the elderly," were identified. A search on YouTube using these terms initially yielded 200 of the most viewed videos for each term. After applying exclusion criteria, 87 videos were included for detailed analysis. Major themes and topics related to anesthesia in geriatric and elderly patients were identified using a pre-determined qualitative thematic analysis method. The usefulness of the videos was assessed using the specially developed Geriatric and Elderly Anesthesia Usefulness Score (GAEUS). The overall quality and reliability of the videos were evaluated using the Global Quality Scale (GQS) and the Modified DISCERN Scale (M-DISCERN), respectively. The average of the quality, reliability, and usefulness scores calculated by the researchers was used for consistency analysis.

Results:
In our study, 48.3% (42) of the videos on geriatric and elderly patients concerning anesthesia on YouTube were created by personal blogs. The quality of the videos was measured using the GQS, with a mean score of 3.34 and a median of 3 (range: 1-5), showing no significant difference according to the video source (p=0.166). Reliability was assessed using the M-DISCERN scale, with a mean score of 3.37 and a median of 3.50 (range: 1-5), again showing no significant difference according to the video source (p=0.097). Usefulness was measured using the GAEUS score, with a mean score of 15.30 and a median of 12.5 (range: 2-63), which showed a significant difference according to the video source (p=0.000). The average duration of videos with low usefulness was 31.59 minutes (range: 5-44), while the average duration of moderately and highly useful videos was 59.37 minutes (range: 19.44-119.05). This duration difference was statistically significant (u=2.569, p=0.010).

Conclusion:
In our study, we examined YouTube videos covering anesthesia topics for geriatric and elderly patients. The highest usefulness scores were obtained from personal blogs; however, all sources generally showed low usefulness. The quality of the videos was assessed using the Global Quality Scale (GQS), and their reliability was evaluated with the Modified DISCERN Scale (M-DISCERN). On both scales, the videos showed moderate performance across all sources. These findings indicate a need for more comprehensive and informative content on YouTube, especially for the education of healthcare professionals and patients. To better address the needs of elderly patients, the richness of content and educational value of these videos should be enhanced.

Keywords: YouTube, Geriatrics, Elderly, Anesthesia, Usefulness

Introduction

Although there is no clear definition of old age in the literature, the world health organization defines individuals aged 60 and older as elderly [1]. By 2030, it is expected that one out of every six people will be over the age of 60 due to the aging population. At the same time, the proportion of elderly

people requiring surgical intervention is increasing globally, and this group accounts for about half of the surgical population in developed countries [2]. Compared with younger individuals, anesthesia-related mortality and morbidity rates are higher in geriatric patients. Advances in modern health have made it possible to perform surgery on elderly people more frequently and have increased the importance of anesthesia applications for this age group [3]. The decrease in physiological functions and increased morbidity in elderly patients disrupt the recovery process after general anesthesia and increase the risk of postoperative complications. The aging process leads to pharmacokinetic and pharmacodynamic changes, affecting the interaction with anesthetic drugs and creating significant differences between age groups in drug responses [4]. For this reason, geriatric patients with surgical requirements require a comprehensive assessment and care from the preoperative period to the postoperative period [5]. International health organizations encourage recommendations for perioperative care for geriatric and elderly patients, but studies in this area are insufficient [6].

Today, health professionals, patients, and their families increasingly use the internet to find solutions to health problems, gain knowledge, and share their experiences [7]. Established in 2005, youtube reaches 95% of internet users and has become an important source of education and information, offering rich video content. Although access to information provided over the internet is easy, the ability of users to effectively evaluate the quality and accuracy of this information is limited. This has led to an increase in studies on the quality and reliability of the information provided, especially on platforms such as youtube [8]. There are many videos on youtube about anesthesia in geriatric and elderly patients. However, comprehensive studies evaluating the content of these videos are not yet available in the literature.

The aim of this study is to assess the quality, reliability, content, and usefulness of anesthesia videos for geriatric and elderly patients on youtube from the perspective of health professionals and patients, using the Global Quality Scale (GQS), the Modified Discern Scale (M-DISCERN), the Geriatric and Elderly Anesthesia Content Scale (GEACS), and the Geriatric and Elderly Anesthesia Usefulness Score (GAEUS). Our hypothesis is that anesthesia videos on youtube for geriatric and elderly patients are of average quality, reliability, content, and usefulness for both health professionals and patients; however, significant differences exist depending on the source of the video.

Method

Our study was designed as an observational research project that evaluates anesthesia videos related to geriatric and elderly patients on YouTube.

Keyword Selection and Video Selection Criteria

Our research was conducted within a systematic framework that included the selection of videos and data collection techniques. Both researchers set a date of January 20, 2024, for the identification and recording of the videos. The cache and cookies were cleared using the incognito mode of the Google Chrome browser. To select the appropriate videos for our study, we first used the terms 'anesthesia in elderly patients,' 'anesthesia in the elderly,' 'geriatric anesthesia,' 'anesthesia in geriatric patients,' and 'anesthesia in the elderly population' through Google Trends, where the most common search trends were deciphered. The most searched terms in the last five years were identified as 'geriatric anesthesia' and 'anesthesia in the elderly.'

Using these keywords, searches were made on YouTube. It was planned to include the top 100 most viewed videos for each search term in the study. The URLs and titles of the collected videos were

31 saved in Microsoft Excel to protect against possible changes over time. After the registration process was completed, the accuracy and completeness of the data were checked. The YouTube videos included in the study were selected based on the highest number of views, and similar previous studies were referenced when determining the sample [8].

Advertisements, repeated content, non-English or voiceless videos, duplicate content, and videos with titles unrelated to the subject matter were excluded from the research.

Analysis of Videos And Detection of Subject Content

32 The titles of the videos, the uploader's name, subscriber counts, and the uploader's country were recorded as basic information. The videos were categorized into three main types based on their source: videos from private and public hospitals and other healthcare institutions were classified as "health institutions"; universities and associations were labeled as "educational institutions"; and healthcare professionals, patients, personal video producers, and independent news channels without a corporate affiliation were categorized as "personal blogs."

5 36 39 The target audiences of the videos were determined to be patients and healthcare professionals based on the information in the channel's "about" section and the video content. Additionally, details such as the year of upload, view counts, days since upload, daily average view rates, video duration, and the number of likes and comments were also recorded. Multi-part videos were evaluated as separate videos for each segment.

Using a similar methodology employed by Henry Robb and colleagues, the main topics and themes related to anesthesia in geriatric and elderly patients on YouTube were identified using a predetermined qualitative thematic analysis method [9].

In parallel with the studies of Byung-Gun Lim and Il-Ok Lee, the main topic headings selected were preoperative, intraoperative, and postoperative management [7]. The researchers reached a consensus on a total of thirty-five themes under these headings, considering their 13-14 years of experience and daily practice.

Under preoperative management, nine themes were identified: definition and epidemiology, pathophysiology, frailty, ASA score and general health assessment, nutritional status, comorbidities, alcohol and substance use, polypharmacy, and depression.

48 In intraoperative management, seventeen themes were identified, including anesthesia management, general anesthesia, spinal anesthesia, epidural anesthesia, peripheral nerve blocks, intubation, ventilation, extubation, monitoring, premedication, induction, anesthetic agents, neuromuscular blockers and antagonists, other pharmacological agents, positioning, fluid management, and blood transfusion. For postoperative management, nine themes were identified: analgesia and postoperative pain, respiratory complications, nausea and vomiting, hypothermia, mobilization, physiotherapy, long-term follow-up and management of chronic conditions, postoperative delirium, and postoperative cognitive dysfunction.

The specific content and scoring system used by Boyang Qu and colleagues was adapted for the YouTube videos in our study as the GEACS [10]. To create the GEACS scale, it was first recorded whether each video contained information related to the predetermined themes. In the videos examined, a score of 0 was assigned if no information was provided on a theme, a score of 1 was given for superficial information, and a score of 2 was awarded for sufficient information. Subsequently, the scores for all themes mentioned in the video were summed to create the GEACS score.

13 Evaluation of the Quality, Reliability, and Usefulness of Videos

6 The overall quality of the videos was evaluated using the GQS and reliability was assessed with the M-DISCERN.

24 The GQS evaluates a video's information flow, completeness of information, and usefulness for patients on a Likert scale from 1 to 5 [11]. This scale rates a score of 1 as low quality (poor flow and incomplete information), 2 as weak quality (limited information), 3 as moderate quality (sufficiently
29 discussed important information), 4 as good quality (good flow and useful information for patients), and 5 as excellent quality (excellent flow and highly useful information) [12]. In our study, videos
7 with GQS scores of 4 or 5 were considered high quality, those with a score of 3 were deemed moderate quality, and scores of 1 or 2 were classified as low quality.

30 The M-DISCERN scoring system is used to assess the reliability of health information regarding various treatment options. This method evaluates the clarity of the videos, source validity, information
8 balance, additional resources, and controversial topics. Each question is scored as 1 for a 'yes' response and 0 for a 'no' response [7]. In our study, M-DISCERN scores above 3 were classified as good, exactly 3 as moderate, and below 3 as poor.

The usefulness score developed by Li M and colleagues was adapted for our study to create the
41 GAEUS. To measure the educational usefulness of the videos, the GEACS scores were combined with the GQS scores to generate the GAEUS score (13). Under the GAEUS scoring system, videos are
1 evaluated on a scale ranging from a minimum of 1 to a maximum of 75 points. Scores between 0-25
35 are classified as minimally useful, 25-50 as moderately useful, and 50-75 as highly useful. Researchers independently evaluated the videos and assigned GQS, M-DISCERN, and GAEUS scores for each video. The final GQS, M-DISCERN, and GAEUS scores for each video were determined by averaging the assigned scores.

14 Primary and Secondary Outcome Measures

The primary outcome measure of our study was determined to be the GAEUS scores. the secondary outcome measures were identified as the GEACS, GQS, and M-DISCERN scores.

Statistical Analysis

3 In the data analysis, IBM SPSS Statistics 25 software (IBM Corp., Armonk, NY) was used. Continuous variables were presented as mean ± standard deviation and median and interquartile range.
19 Categorical variables were given as numbers and percentages. For the reliability analysis of the study, Cronbach's Alpha value was calculated and found to be 0.894, showing high consistency. For validity and reliability assessments, skewness and kurtosis analyses were performed; the accepted range for normality was determined as ±1.5. The maximum value obtained in our results was -1.076, indicating that the scales were objectively valid and reliable. Cohen's kappa coefficient was taken for the consistency of the observers scoring the videos, and values of 0.758 for the GQS score showed that internal consistency was achieved. According to the Kendall's tau correlation performed, the GQO-
25 usefulness score (r:0.367; p:0.000) and MDO-usefulness score (r:0.380; p:0.000) were statistically
9 significant and highly correlated. Descriptive analyses were initiated. Mann-Whitney U test was used

to compare group differences between continuous variables. Tukey's test was used for the analysis of within-group changes over time. Chi-square test was used for comparisons between categorical variables and groups. In all analyses, $p < 0.05$ was considered statistically significant.

Findings

Initially, 100 videos related to geriatric anesthesia and 83 videos related to anesthesia in the elderly were found on YouTube. The study began with 183 videos; however, 96 videos were excluded from the study due to 20 shorts, 11 advertisements, 9 non-English videos, 7 unrelated titles, 6 veterinary-related videos, 2 voiceless videos, and 41 duplicates. The remaining 87 videos were examined in detail (Figure 1).

Figür 1. Flow Chart

In our study, the descriptive characteristics of YouTube videos on anesthesia for geriatric and elderly patients were presented in Table 1. According to the results of our research, a significant portion of the videos, 48.3% (42 videos), was created by personal blogs. Content uploaded by educational institutions accounted for 36.8% (32 videos), while videos uploaded by healthcare facilities comprised 14.9% (13 videos). Most of the information in the videos was provided by doctors (94.3%, 82 videos).

Regarding the geographical distribution of the videos, the highest proportion came from unknown sources at 33.3% (29 videos), while the most videos were uploaded from the United States, accounting for 32.2% (28 videos).

The average duration of the examined videos was 35.42 minutes, with a median duration of 15 minutes (minimum 1 minute, maximum 134 minutes). The average number of likes was 114.93, while the median number of likes was 7. The average duration that the videos remained on the platform was measured at 1139.03 days, with a median of 908 days. In terms of quality and reliability assessment, the GQS score averaged 3.34 with a median of 3 (range 1-5), and the M-DISCERN score averaged 3.37 with a median of 3.50 (range 1-5). The GEACS score averaged 11.95 with a median value of 9 (range 0-58), while the GAEUS score averaged 15.30 with a median value of 12.5 (range 2-63).

Table 1. Descriptive Characteristics of Anesthesia Video Content in Geriatric and Elderly Patients on YouTube

In our study, thirty-five themes were evaluated under three main topic headings related to anesthesia videos for geriatric and elderly patients on YouTube (Table 2).In preoperative management, a total of nine sub-themes were addressed. According to the content distribution, healthcare institutions produced approximately 14.7% of the content with 22 entries, educational institutions contributed 48.0% with 72 entries, and personal blogs accounted for 38.7% with 58 entries. During this period, the topic theme with the most information across all sources was Pathophysiology. Educational institutions provided significantly higher information on the ASA score and general health assessment topic compared to other sources ($p = 0.048$).

In intraoperative management, seventeen sub-themes were examined. In the content distribution, healthcare institutions contributed 13.8% with 34 entries, educational institutions contributed 41.5% with 102 entries, and personal blogs accounted for 52.9% with 130 entries. The topic theme with the most information across all sources during this period was Anesthesia Management. Educational

institutions provided significantly higher information on the endotracheal intubation topic compared to other sources (p= 0.024).

In postoperative management, nine different sub-themes were analyzed. Healthcare institutions presented 12.2% with 14 entries, educational institutions presented 45.2% with 52 entries, and personal blogs presented 44.3% with 51 entries. During this period, the topic with the most information from educational institutions was Postoperative Delirium, with 37.5% (12 entries), and from personal blogs, it was also Postoperative Delirium with 35.7% (15 entries); from healthcare institutions, the topic with the most information was Postoperative Cognitive Dysfunction at 30.8% (4 entries).

In the analysis of the usefulness scores of videos related to anesthesia practices for geriatric and elderly patients, healthcare institutions provided content in the low usefulness category at 92.3%, educational institutions at 87.5%, and personal blogs at 83.3%. In the moderate usefulness category, healthcare institutions provided 7.7%, educational institutions 9.4%, and personal blogs 14.3%. In the high usefulness category, educational institutions and personal blogs provided very low content (3.1% and 2.4%, respectively), while healthcare institutions provided no content at all. No statistically significant difference was found between the GAEUS scores of the videos based on the sources (p=0.899).

Table 2. Comparison of Themes And Usefulness Scores of Anesthesia Videos in Geriatric and Elderly Patients on YouTube According to the Video Source

The usefulness scores of video content on anesthesia topics for geriatric and elderly patients on YouTube were compared in Table 3. The average duration of videos providing low usefulness was determined to be 31.59 minutes (range 5-44), while the average duration of videos providing moderate and high usefulness was 59.37 minutes (range 19.44-119.05). This difference in duration was statistically significant (U=2.569, P=0.010).

Videos providing low usefulness were published an average of 1182.37 days ago, while those providing moderate and high usefulness were published an average of 868.17 days ago; however, this difference in duration was not statistically significant (U=0.659, P=0.510). Videos providing low usefulness were viewed an average of 15052 times, while those providing moderate and high usefulness were viewed an average of 990.33 times; the difference in view counts was not statistically significant (U=0.222, P=0.825).

In terms of view rate, videos providing low usefulness were measured at an average of 7.58, while this rate for videos providing moderate and high usefulness was determined to be 1.07; however, this difference was not statistically significant (U=0.191, P=0.848). Videos providing low usefulness received an average of 129.99 likes, while those providing moderate and high usefulness received an average of 20.83 likes; this difference in the number of likes was also not statistically significant (U=0.735, P=0.463).

Regarding the number of comments, videos providing low usefulness had an average of 13.73 comments, while the average for videos providing moderate and high usefulness was 1.25; this difference was not statistically significant (U=0.573, P=0.567). The average M-DISCERN score for videos providing low usefulness was measured at 3.31, while for videos providing moderate and high usefulness, it was 3.83; however, no statistical difference was observed (U=1.352, P=0.177).

Table 3. Comparison of Anesthesia Videos in Geriatric and Elderly Patients on YouTube according to usefulness scores

A comparison of video content on anesthesia topics for geriatric and elderly patients on YouTube was conducted based on the uploader source (Table 4). GQO for videos from healthcare institutions was 3 (ranging from 2 to 5), while videos from educational institutions received a median score of 4 (ranging from 3 to 4.88), and videos from personal blogs scored 3 (ranging from 2 to 4). The GQO scores of the videos did not show a statistically significant difference based on the uploader source (p=0.166).

The M-DISCERN score revealed that healthcare institutions and personal/other sources had similar median scores (3), while educational institutions achieved a higher median value (4). However, the differences in M-DISCERN scores were not statistically significant (p=0.097).

For videos from healthcare institutions, the median score for the GEACS was determined to be 5 (range 4-14), while for educational institutions it was 8.50 (range 2.5-13.75), and for personal/other sources, it was 10.5 (range 6-16.50). This difference was found to be statistically significant (p=0.000).

For the GAEUS, the median usefulness score for videos from healthcare institutions was 9.50 (range 7.5-17.5), from educational institutions it was 12.5 (range 7-18), and from personal blogs it was 13.75 (range 8-20). These differences were statistically significant (p=0.000).

Table 4. Comparison of Quality, Reliability, Scope And Usefulness of Video Content on Anesthesia in Geriatric And Elderly Patients on Youtube According To The Uploader's Source

Discussion

Our study is, to our knowledge, the first to evaluate the quality, reliability, content, and usefulness of anesthesia videos for geriatric and elderly patients on YouTube. In our study, we hypothesized that the anesthesia videos for geriatric and elderly patients on YouTube would be of moderate quality, reliability, content, and usefulness but would show significant differences depending on the sources. We assessed the usefulness of the videos using the GAEUS scoring system and found that they generally exhibited low usefulness. The highest GAEUS scores were obtained from personal blogs, followed by educational institutions and healthcare organizations. The quality of the videos was measured using the GQS and their reliability was assessed with the DISCERN scale. Contrary to our hypothesis, we found no significant differences between the sources, as both scales indicated that the videos were of moderate quality. When measuring the thematic content scope with the GEACS, we

determined that the broadest content was created by personal blogs. The most frequently addressed theme across all sources was anesthesia methods. The themes of ASA scores and general health assessments provided significantly more information in educational institutions compared to other sources.

45 Our research is, to our knowledge, the first study that evaluated the quality, reliability, content, and usefulness of anesthesia videos for geriatric and elderly patients on YouTube. In our study, we hypothesized that the anesthesia videos for geriatric and elderly patients on YouTube would be at a moderate level in terms of quality, reliability, content, and usefulness, but would show significant differences depending on the sources. We assessed the usefulness of the videos using the GAEUS and found that they generally exhibited low usefulness. The highest GAEUS scores were obtained from personal blogs, followed by educational institutions and healthcare organizations. 21 In our study, where we measured the quality of the videos with the GQS and their reliability with the M-DISCERN, we found that the videos were of moderate quality on both scales, as we hypothesized, but contrary to our hypothesis, no significant differences were found between the sources. When the thematic content scope was measured with the GEACS, we determined that the broadest content was created by personal blogs. The most frequently addressed theme across all sources was anesthesia methods. The themes of ASA scores, general health assessments, and intubation were significantly more covered in educational institutions compared to other sources [13].

Our study comprehensively analyzed 87 YouTube videos that have been viewed a total of 1.14 million times and contain about 51 hours of content. When we look at the geographical distribution of video sources, the most content was created from the USA in our study and showed a similar distribution to the literature [15]. In our research, although similar results were obtained with the literature in terms of 18 the educational usefulness of videos, the quality and reliability scores of our videos were found to be higher than the literature. Similar studies in the literature may explain the fact that although videos are 5 mostly based on personal blog sources, our quality and reliability scores are high due to the fact that the total of videos produced by health institutions and educational groups in our study is higher than personal blogs and these institutions produce higher quality content [14].

In our research, videos prepared mostly by doctors and health professionals achieved high quality scores above what is expected in the literature. We think that the fact that videos from other sources, such as personal blogs, also received high GAEUS scores may have been due to the higher quality of 46 these contents than expected. In this context, we think that the fact that the quality and reliability scores of our videos are higher than the literature can be associated with the high degree of professionalism of the content presented. Although the educational usefulness of our videos is similar to that reported in the literature, the fact that their overall quality and reliability levels are lower may be due to our specially developed and detailed GEACS scoring system, which evaluates 35 different themes separately. This system measures the educational depth and content breadth of videos according to the number of themes. Consistent with studies in the literature, our videos were found to have low topic content and resulted in low educational usefulness scores [16]. Our study has found a significant relationship between video duration and usefulness scores. The increase in usefulness scores with the extension of video duration has also been observed in past studies [17]. We think that by increasing the duration of anesthesia videos in geriatric and elderly patients, the scope of subject content can be expanded, so their usefulness can increase. We did not find a relationship between 15 viewer interactions, such as the number of views, likes and comments of videos, and the usefulness of

videos. This result supports previous studies [18]. As a result, we also think that these audience interactions should not be used for usefulness.

In our study, the most frequently covered themes among all video sources were anesthesia management and general anesthesia, while regional anesthesia and especially intubation were less frequently covered. On the other hand, a study conducted on YouTube shows that the topics related to regional anesthesia, intubation and general anesthesia are examined the most in perioperative anesthesia videos. The frequency of themes in our study does not align with the titles more commonly used in other studies analyzing YouTube videos. [19]. This discrepancy may be due to the low popularity of anesthesia videos aimed at geriatric and elderly patients. Although we selected the videos we took into the study from the most watched videos, the viewing rates used in many studies as an indicator of popularity in the literature are quite low in our study [20]. Additionally, a recent study showed that aging and death were the least popular topics among 10 major global health education themes. These results supported our theory that the popularity of anesthesia videos is low in geriatric and elderly patients [21].

With the aging population, the difficulties and complications faced by anesthesiologists are increasing. Elderly patients often face various health problems, such as multiple system disorders. this makes it mandatory to prepare customized anesthesia plans according to the individual needs of patients [22]. Many studies emphasize the importance of implementing the published guidelines for the perioperative care of the elderly [23]. However, various studies show that compliance with these guidelines is low [24]. In particular, video content providers should continue to produce more video content aimed at both healthcare professionals and patients based on current guidelines on disseminating accurate information about geriatric and elderly patients. We think that YouTube, which is a particularly popular video sharing site, can make an important contribution to meeting the need for useful information for this group. In addition, we also agree with the views that the academic incentives proposed in previous studies can contribute to the production of high-quality content on YouTube, and it may be useful to create a peer-reviewed section to disseminate accurate information [25].

Our research has some important limitations. Firstly, the usefulness scoring system that we use to evaluate videos is not a generally accepted or verified method and is largely subjective. Second, the keywords we used in the video search process were assumed to be what a typical user would prefer, which could create potential bias. Because different users or situations may use various keywords and this may affect the results obtained. Third, our study only focused on English content, which may result in ignoring relevant videos in other languages. In addition, videos are sorted by the number of views on YouTube™, these ranking criteria may affect the results obtained. Finally, YouTube search results are constantly changing; adding new videos or Deconstructing existing ones can cause the results to change over time.

Results

In our study, we examined YouTube videos covering anesthesia topics for geriatric and elderly patients. The highest usefulness scores were obtained from personal blogs; however, all sources generally showed low usefulness. The overall quality of the videos was evaluated using the Global Quality Scale (GQS), and their reliability was assessed with the Modified DISCERN Scale. On both scales, the videos performed at a moderate level across all sources. These findings indicate the need for more comprehensive and informative resources on the YouTube platform, especially for the education of healthcare professionals and patients. To better meet the needs of elderly patients, the richness of content and educational value of the videos should be improved.

Resources

1. Guidet B, Vallet H, Flaatten H, Joynt G, Bagshaw SM, Leaver SK, Beil M, Du B, Forte DN, Angus DC, Sviri S, De Lange D, Herridge MS, Jung C. The trajectory of very old critically ill patients. Intensive Care Med. 2024 Feb;50(2):181-194. https://doi.org/10.1007/s00134-023-07298-z. Epub 2024 Jan 18. PMID: 38236292.

2. Bettelli G. Geriatric anesthesia: Demographics, epidemiology, state of the art at international level, educational needs, and future perspectives. Saudi J Anaesth. 2023 Oct-Dec;17(4):467-473. https://doi.org/10.4103/sja.sja_411_23. Epub 2023 Aug 18. PMID: 37779572; PMCID: PMC10540985.

3. Al Harbi MK, Alshaghroud SM, Aljahdali MM, Ghorab FA, Baba F, Al Dosary R, Bahadeq M. Regional anesthesia for geriatric population. Saudi J Anaesth. 2023 Oct-Dec;17(4):523-532. https://doi.org/10.4103/sja.sja_424_23. Epub 2023 Aug 18. PMID: 37779559; PMCID: PMC10540989.

4. Alghamdi AS, Almuzayyen H, Chowdhury T. The elderly in the post-anesthesia care unit. Saudi J Anaesth. 2023 Oct-Dec;17(4):540-549. https://doi.org/10.4103/sja.sja_528_23. Epub 2023 Aug 18. PMID: 37779571; PMCID: PMC10540998.

5. Aceto P, Antonelli Incalzi R, Bettelli G, Carron M, Chiumiento F, Corcione A, Maggi S, Montorsi M, Pace MC, Petrini F, Tommasino C, Trabucchi M, Volpato S. Perioperative management of elderly patients (PRIME): recommendations from an Italian intersociety consensus. Aging Clin Exp Res. 2020 Sep;32(9):1647-1673. https://doi.org/10.1007/s40520-020-01624-x. Epub 2020 Jul 10. Erratum in: Aging Clin Exp Res. 2020 Sep;32(9):1907. https://doi.org/10.1007/s40520-020-01701-1. PMID: 32651902; PMCID: PMC7508736.

6. American Geriatrics Society Expert Panel on Postoperative Delirium in Older Adults. Postoperative delirium in older adults: best practice statement from the American Geriatrics Society. J Am Coll Surg. 2015 Feb;220(2):136-48.e1. https://doi.org/10.1016/j.jamcollsurg.2014.10.019. Epub 2014 Nov 14. PMID: 25535170.

7. Kartufan FF, Bayram E. The evaluation of YouTube™ videos pertaining to intraoperative anaesthesia awareness: A reliability and quality analysis. Cureus. 2023 Mar 8;15(3)

. https://doi.org/10.7759/cureus.35887. PMID: 37033592; PMCID: PMC10081863.

8. Duran MB, Kizilkan Y. Quality analysis of testicular cancer videos on YouTube. Andrologia. 2021;53(8) https://doi.org/10.1111/and.14118

9. Robb H, Scrimgeour G, Boshier P, Przedlacka A, Balyasnikova S, Brown G, Bello F, Kontovounisios C. The current and possible future role of 3D modelling within oesophagogastric surgery: a scoping review. Surg Endosc. 2022 Aug;36(8):5907-5920. https://doi.org/10.1007/s00464-022-09176-z. Epub 2022 Mar 11. PMID: 35277766; PMCID: PMC9283150

10. Qu B, Kang B, Chen X, Ao Y, Wang L, Cui W. YouTube as a source of information on preventing the use of valproic acid in women during pregnancy. BMC Public Health. 2023 Jun 23;23(1):1225. https://doi.org/10.1186/s12889-023-16036-5. PMID: 37353789; PMCID: PMC10290355.

11. Aurlene N, Shaik SS, Dickson-Swift V, Tadakamadla SK. Assessment of usefulness and reliability of YouTube™ videos on denture care. Int J Dent Hyg. 2024 Feb;22(1):106-115. https://doi.org/10.1111/idh.12771. Epub 2023 Oct 4. PMID: 37793081.

12. Ali Baig S, Malhotra K, Banerjee AJ, Kowsik M, Kumar K, Rahman F, Batul SS, Saiyed MF, Venkatesh V, Viswanath Iyer P, Kempegowda P. Assessment of the quality, content, and reliability of YouTube® videos on diabetes mellitus and polycystic ovary syndrome: a systematic review with cross-sectional analysis comparing peer-reviewed videos. Endocr Connect. 2024 Jun 28;13(7)

. https://doi.org/10.1530/ec-24-0059. PMID: 38856005; PMCID: PMC11227060.

13. Li M, Yan S, Yang D, Li B, Cui W. YouTube™ as a source of information on food poisoning. BMC Public Health. 2019 Jul 16;19(1):952. https://doi.org/10.1186/s12889-019-7297-9. PMID: 31311523; PMCID: PMC6636170.

14. Javidan A, Nelms MW, Li A, Lee Y, Zhou F, Kayssi A, Naji F. Evaluating YouTube as a Source of Education for Patients Undergoing Surgery: A Systematic Review. Ann Surg. 2023 Oct 1;278(4)

. https://doi.org/10.1097/SLA.0000000000005892. Epub 2023 May 5. PMID: 37144414

15. Parmar UPS, Ichhpujani P, Chahal R, Singh RB. Reliability of Ahmed glaucoma valve surgical videos for educational purposes. Int Ophthalmol. 2023 Sep;43(9):3425-3432. https://doi.org/10.1007/s10792-023-02734-x. Epub 2023 May 16. PMID: 37191927; PMCID: PMC10185961.

16. Osman W, Mohamed F, Elhassan M, et al. Is YouTube a reliable source of health-related information? A systematic review. BMC Med Educ 22, 382 (2022). https://doi.org/10.1186/s12909-022-03446-z

17. Czerwonka N, Reynolds AW, Saltzman BM, Alexander F, Trofa DP, Ahmad CS. The Quality of YouTube Content on Ulnar Collateral Ligament Injuries Is Low: A Systematic Review of Video Content. Arthrosc Sports Med Rehabil. 2023 Aug 8;5(6):100769. https://doi.org/10.1016/j.asmr.2023.100769. PMID: 38155764; PMCID: PMC10753170.

18. MacLeod MG, Hoppe DJ, Simunovic N, Bhandari M, Philippon MJ, Ayeni OR. YouTube as an information source for femoroacetabular impingement: a systematic review of video content. Arthroscopy. 2015 Jan;31(1):136-42. https://doi.org/10.1016/j.arthro.2014.06.009. Epub 2014 Aug 20. PMID: 25150406.

19. Nelms MW, Javidan A, Chin KJ, Vignarajah M, Zhou F, Tian C, Lee Y, Kayssi A, Naji F, Singh M. YouTube as a source of education in perioperative anesthesia for patients and trainees: a systematic review. Can J Anaesth. 2024 Jun 20. English. https://doi.org/10.1007/s12630-024-02791-5. Epub ahead of print. PMID: 38902576

20. Kwak D, Park JW, Won Y, et al Quality and reliability evaluation of online videos on carpal tunnel syndrome: a YouTube video-based study BMJ Open 2022;12

. https://doi.org/10.1136/bmjopen-2021-059239

21 Campbell IH, Rudan I. A systematic analysis of online public engagement with 10 videos on major global health topics involving 229 459 global online viewers. J Glob Health. 2020 Jun;10(1):010903. https://doi.org/10.7189/jogh.10.010903. PMID: 32257172; PMCID: PMC7101210.

22. Van Zundert A, Gatt SP, van Zundert TC. Prevention and treatment of noncognitive complications. Saudi J Anaesth. 2023 Oct-Dec;17(4):557-565. https://doi.org/10.4103/sja.sja_329_23. Epub 2023 Aug 18. PMID: 37779568; PMCID: PMC10540984.

23. Birkelbach O, Mörgeli R, Spies C, Olbert M, Weiss B, Brauner M, Neuner B, Francis RCE, Treskatsch S, Balzer F. Routine frailty assessment predicts postoperative complications in elderly patients across surgical disciplines - a retrospective observational study. BMC Anesthesiol. 2019 Nov 7;19(1):204. https://doi.org/10.1186/s12871-019-0880-x. PMID: 31699033; PMCID: PMC6839249.

24. Clark C, Bennett E, Foo I. Adherence to published guidelines for perioperative care of the elderly: a survey of Scottish anaesthetic departments. Perioper Med (Lond). 2022 Jul 5;11(1):26. https://doi.org/10.1186/s13741-022-00258-z. PMID: 35787738; PMCID: PMC9253247.

Table 1. Descriptive Characteristics of Anesthesia Video Content in Geriatric and Elderly Patients on YouTube

Content information of the videos	Features	*N* (%)	
Video source	Health institution	13 (%14,9)	
	Educational institution	32 (%36,8)	
	Personal/Blog	42 (%48,3)	
The profession of the uploader	Doctor	82 (%94,3)	
	Other	5 (%5,7)	
Uploaded country	Usa	28 (%32,2)	
	India	12 (%13,8)	
	Other	18 (%20,7)	
	Unknown	29 (%33,3)	
Technical information of the videos	Mean±s.s.	Median (min.-max.)	Total
Video duration (minutes)	35,42±40,38	15 (1-134)	3082 min
Number of likes	114,93±519,34	7 (0-4500)	9998
Number of comments	12,01±83,51	0 (0-774)	14058
The time elapsed from the day of publication to today (day)	1139,03±909,55	908 (12-4552)	99096 day
Number of views	13112,86±59477,37	325 (8-498,301)	1140819
View rate (number of views/time spent)	6,59±26,84	0,46 (0,02-206,96)	520,87
M DISCERN	3,37±1,17	3,50 (1-5)	
GQO	3,34±1,16	3 (1-5)	
GEACS (1 -70)	11,95±11,29	9 (0-58)	
GAEUS (2 – 75)	15,30±11,67	12,5 (2-63)	

Table 2. Comparison of Themes And Usefulness Scores of Anesthesia Videos in Geriatric and Elderly Patients on YouTube According to the Video Source

Main theme	Sub-theme	Health institutions (n=13)	Educational institutions (n=32)	Personal/ Blog (n=42)	p
Preoperative management	Definition epidemiology	3 (%23.1)	8 (%25)	12 (%28,2)	0.901
	Pathophysiology	5 (%38.5)	18 (%56.3)	18 (%42.9)	0.413
	Fragility	4 (%30.8)	11 (%34.4)	6 (%14.3)	0,112
	ASA Score - General health values assessment	0	5 (%15.6)	1 (%2.4)	0.048*
	Nutrition	3 (%23.1)	4 (%12.5)	2 (%4.8)	0.146
	Concomitant diseases or comorbidity	3 (%23.1)	14 (%43.8)	15 (%35.7)	0.419
	Substance use	1 (%7.7)	1 (%3.1)	0	0.250
	Polypharmacy	2 (%15.4)	6 (%18.8)	4 (%9.5)	0.514
	Depression	1 (%7.7)	3 (%9.4)	3 (%7.1)	0.939
Introperative management	Anesthesia management	9 (%69.2)	22 (%68.8)	36 (%85.7)	0.176
	General anesthesia	7 (%53,8)	17 (%53.1)	29 (%69)	0.324
	Spinal anesthesia	3 (%23.1)	4 (%12.5)	6 (%14.3)	0.657
	Epidural anesthesia	1 (%7.7)	2 (%6.3)	3 (%7.1)	0.981
	Peripheral nerve block	2 (%15.4)	5 (%15.6)	4 (%9.5)	0,699
	Endotracheal Entubation	0	7 (%21,9)	2 (%4,8)	0.024*
	Ventilasyon	2 (%15.4)	8 (%25)	10 (%23.8)	0.773
	Extubation	1 (%7.7)	1 (%7.7)	2 (%4.8)	0.801
	Monitoring	5 (%38.5)	19 (%)59.4	26 (%61.9)	0.315

	Premedication	0	4 (%12,5)	6 (%14,3)	0.360
	Induction	0	3 (%9.4)	3 (%7.1)	0.529
	Anesthetic agents	0	5 (%15.6)	4 (%9.5)	0.288
	Neuromuscular blockers and their antagonists	0	2 (%6.3)	5 (%11.9)	0.346
	Other pharmacological agents	4 (%30.8)	11 (%34.4)	22 (%52.4)	0.195
	Position	0	4 (%12.5)	9 (%21.9)	0.148
	Fluid management	0	6 (%18,8)	8 (%19)	0.231
	Blood transfusion	0	2 (%6.3)	1 (%2.4)	0.506
Postoperative management	Respiratory complications	3 (%23.1)	10 (%31.3)	11 (%26.2)	0.824
	Nausea and vomiting	0	2 (%6.3)	1 (%2.4)	0.506
	Hypothermia	1 (%7.7)	4 (%12.5)	5 (%11.9)	0.894
	Mobilization	1 (%7.7)	1 (%3.1)	1 (%2.4)	0.651
	Physiotherapy	2 (%15.4)	5 (%15.6)	1 (%2.4)	0.105
	Analgesia and postoperative pain	1 (%7.7)	7 (%21.9)	10 (%23.8)	0.446
	Long-term follow-up and management of chronic conditions	0	2 (%6.3)	1 (%2.4)	0.506
	Postoperative delirium	2 (%15.4)	12 (%37.5)	15 (%35.7)	0.326
	Postoperative cognitive dysfunction	4 (%30.8)	7 (%21.9)	15 (%35.7)	0.435
Total	35	70 %13.08	226 %42.24	239 %44.67	
GAEUS (0 25) low benefit		12 (%92.3)	28 (%87.5)	35 (%83.3)	0.899
GAEUS (26-50) moderate benefit		1 (%7.7)	3 (%9.4)	6 (%14.3)	
GAEUS (50 75) high benefit		0	1 (%3.1)	1 (%2.4)	

p < 0.05 is statistically significant (*).

Table 3. Comparison of Anesthesia Videos in Geriatric and Elderly Patients on YouTube according to usefulness scores

Technical specifications	GAEUS (0-25) low benefit	GAEUS (26-75) medium and high benefit	U	P
Video Duration (Minutes)	13 (5-44)	38 (19.44-119.05)	2.569	0.010*
The time elapsed from the day of publication to today (day)	1182.37 (605-1503)	868.17 (647-1056)	0.659	0.510
Number of views	296 (99-2016)	399 (184.50-651.75)	0.222	0.825
View rate (number of views/time spent)	0.44 (0.11-1.89)	0.48 (0.28-0.76)	0.191	0.848
Number of likes	6 (1-24)	9 (5.25-13.25)	0.735	0,463
Number of comments	0 (0-1)	0 (0-1,75)	0.573	0.567
M DISCERN	3 (2-4.5)	4 (3.13-4.5)	1.352	0.177

$p < 0.05$ is statistically significant (*).

Table 4. Comparison of Quality, Reliability, Scope And Usefulness of Video Content on Anesthesia in Geriatric And Elderly Patients on Youtube According To The Uploader's Source

	Health institution (n=13)	Educational institution (n=32)	Personal/Blog (n=42)	H	P
GQO	3 (2-5)	4 (3-4,88)	3 (2-4)	3.589	0.166
M DISCERN	3 (2-5)	4 (3-4.5)	3 (2-4)	4.667	0.097
GEACS	5 (4-14)	8.50 (2.5-13.75)	10.5 (6-16.50)	30.926	0.000*
GAEUS	9.50 (7,5-17.5)	12.5 (7-18)	13.75 (8-20)	30.833	0.000*

$p < 0.05$ is statistically significant (*).

Figür.1 Flow Chart

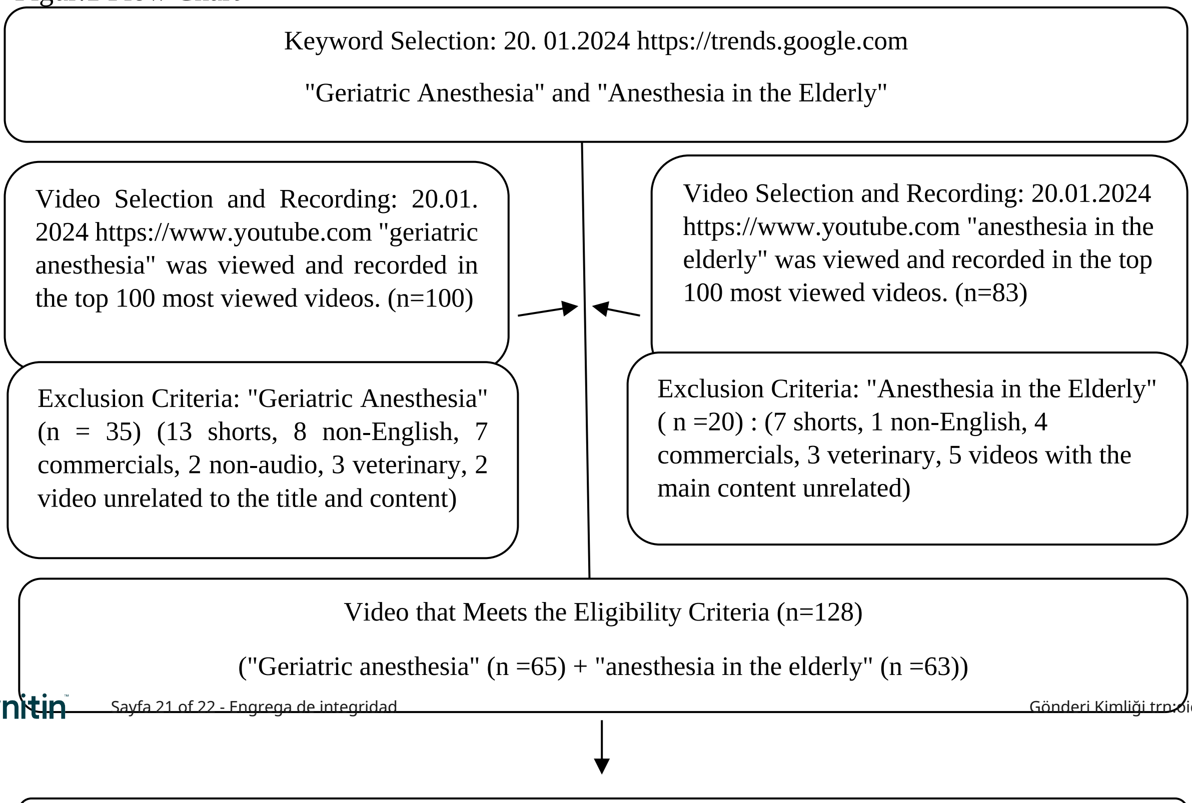

The Total Number of Videos Included in the Study (n= 87)
